# Evaluating the Role of Basiliximab Induction in Simultaneous Liver–Kidney Transplantation: A Multicenter Propensity-Score-Matched Analysis

**DOI:** 10.3390/antib14040091

**Published:** 2025-10-28

**Authors:** Avery Koi, Trine Engebretsen, Alfred S. Lea, Daniel Arango, Heather L. Stevenson, Michael L. Kueht

**Affiliations:** 1 John Sealy School of Medicine, The University of Texas Medical Branch, Galveston, TX 77555, USA; 2 Division of Transplant and Hepatobiliary Surgery, Department of Surgery, The University of Texas Medical Branch, Galveston, TX 77555, USA; 3 Division of Infectious Disease, Department of Medicine, The University of Texas Medical Branch, Galveston, TX 77555, USA; 4 Department of Anesthesiology, The University of Texas Medical Branch, Galveston, TX 77555, USA; 5 Division of Transplant Pathology, Department of Pathology, The University of Texas Medical Branch, Galveston, TX 77555, USA

**Keywords:** combined liver-kidney transplant, simultaneous liver-kidney transplant, basiliximab, induction immunosuppression, retrospective case-control study, TriNetX database

## Abstract

Introduction: Simultaneous liver–kidney (SLK) transplant recipients are considered at lower immunologic risk than kidney-alone recipients, so steroid-only induction is often used. However, some centers continue to include basiliximab induction in their protocols. This study compared graft and infectious outcomes in SLK recipients receiving basiliximab (Bas) induction versus those without basiliximab (No Bas). Methods: Using TriNetX, we conducted a retrospective, propensity-score-matched study of SLK recipients comparing 3-, 6-, and 12-month graft and infectious outcomes. Patients receiving alemtuzumab or anti-thymocyte globulin were excluded; steroid induction was permitted but not required in either cohort. Maintenance immunosuppression included tacrolimus, mycophenolate, and prednisone. Cohorts were matched on 71 variables, including demographics, disease etiology, severity markers, and cPRA. Results: After matching, 292 patients were included per cohort (mean age 56.9 ± 10.1 years; 61% male). Kidney and liver rejection rates were similar. The No Bas cohort had more liver biopsies (25.5% vs. 18.2% at 1 year, *p* = 0.04). Kidney biopsy, graft failure, re-transplantation, delayed graft function, and mortality were comparable. Liver primary non-function was more frequent in Bas (2.8% vs. 0.4%, *p* = 0.04). The No Bas cohort had higher CMV at 3 months (13.4% vs. 6.7%, *p* = 0.008) and higher EBV at all time points (4.0% vs. 0.4% at 1 year, *p* = 0.004). Conclusions: SLK recipients without basiliximab induction had comparable rejection outcomes but more viral infections, potentially from greater steroid exposure, and more liver biopsies, which may reflect higher clinical suspicion for rejection or incomplete capture of rejection events in EMR data.

## 1. Introduction

Worldwide, simultaneous liver–kidney (SLK) transplantation has become an increasingly utilized treatment modality for patients with both end-stage liver disease and chronic kidney disease or sustained acute kidney injury. The number of SLKs performed has steadily increased over the past two decades, although the frequency varies by region, and there are different allocation criteria among countries. In the United States, there has been an increase in SLKs performed since the introduction of the Model for End-Stage Liver Disease (MELD) scoring system in 2001, which incorporates renal function into the prioritization of liver transplant candidates. In 2020, SLKs accounted for 8.4% of all liver transplants in the U.S. [1]. Despite this increase in SLKs being performed, the optimal immunosuppression strategy for SLK recipients remains incompletely defined. SLK transplantation is generally considered to be immunologically lower risk, as the liver allograft exerts an immunoprotective effect on the kidney allograft, particularly when compared to kidney transplantation alone [2,3]. Nevertheless, immunosuppression remains a critical component of SLK transplantation, and no universal consensus exists regarding the optimal regimen.

Induction therapy plays a key role in reducing the risk of acute rejection in the post-transplant period. The most commonly used agents for induction include T-lymphocyte-depleting agents, such as anti-thymocyte globulin, the B–cell–depleting monoclonal antibody alemtuzumab, and interleukin-2 (IL-2) receptor antagonists, such as basiliximab, which are often administered in conjunction with high-dose steroids. The use of induction immunosuppression agents is well-established in kidney transplantation to reduce acute rejection. Anti-thymocyte globulin is more potent and preferred for patients with higher immunologic risk, while basiliximab is more commonly used in those with low-to-moderate immunologic risk. In liver transplantation, the benefits of these induction agents are widely debated, as the liver is generally more resistant to rejection than the kidney; consequently, many centers do not use either anti-thymocyte globulin or basiliximab in liver transplant recipients. Because SLK recipients encompass both organ types, the optimal induction strategy remains unclear; however, the immunoprotective effect of the liver generally renders SLK transplantation lower immunologic risk than kidney transplantation alone. A recent analysis of the Organ Procurement and Transplant Network registry found that, among SLK recipients between 1996 and 2016, 18% received T-cell depletion induction, 32% received an IL-2 receptor antagonist, and 50% received no induction, highlighting the fact that no standardized induction regimen currently exists for SLK recipients [4]. This variability in practice underscores the need to examine the efficacy of specific induction agents, such as basiliximab, in SLK transplantation.

In liver-only transplantation, the benefit of basiliximab in preventing rejection remains controversial, as some studies have found no additional advantage over steroid-only protocols. However, basiliximab has been shown to be beneficial when used in conjunction with delayed tacrolimus protocols, reducing the risk of acute kidney injury and decline in renal function without increasing the risk of rejection [5]. In kidney transplantation, basiliximab has an established role and is effective in preventing acute rejection in select patients [6,7,8]. There are very few studies examining the role of basiliximab and other induction agents in SLK recipients. One study found no benefit of basiliximab over steroid-only protocols, while another showed that induction immunosuppression had no impact on overall patient and allograft survival [4,9]. Another multicenter analysis comparing no induction to anti-thymocyte globulin and basiliximab found no benefit for graft or patient survival. Instead, it revealed that induction with anti-thymocyte globulin increased mortality in SLK recipients compared to no induction [10]. Despite these few studies showing induction immunosuppression may not be warranted in SLK recipients, many centers still use basiliximab and other induction agents as part of their induction protocol.

Given the importance of preventing rejection in both grafts while also avoiding over-immunosuppression, this study aims to further investigate the role of basiliximab as an induction agent in SLK transplantation. In this study, we evaluate the impact of basiliximab versus no basiliximab induction on graft, patient, and infectious outcomes at 3, 6, and 12 months post-transplant, using real-world patient data from multiple centers.

### Aims

The primary objective of this paper is to evaluate the impact of basiliximab on graft and patient outcomes, including rejection, graft failure, and mortality, at 3, 6, and 12 months post-SLK transplant. The secondary objective of this paper is to evaluate infectious outcomes, including opportunistic viral infections, pneumonia, sepsis, and pyelonephritis at 3, 6, and 12 months post-SLK transplant.

## 2. Materials and Methods

### 2.1. Data Source and Collection

This study utilized the TriNetX (Cambridge, MA, USA) “US Collaborative Network” database to identify first-time simultaneous liver–kidney transplant (SLK) recipients. This database contains data from ~130 million de-identified patient electronic medical records (EMR) from ~64 healthcare organizations from across the United States.

TriNetX aggregates data directly from the EMRs of participating healthcare organizations. Data feeds are refreshed on a regular basis (typically weekly to monthly) and include patient demographics, diagnoses, procedures, laboratory values, and medications. To ensure consistency across sites, TriNetX standardizes data by mapping them to widely used coding systems, including the International Classification of Diseases, Ninth and Tenth Revisions (ICD-9/10), Current Procedural Terminology (CPT), ICD-10 Procedure Coding System (ICD-10-PCS), Logical Observation Identifier Names and Codes (LOINC) for laboratory values, and RxNorm for medications. This process allows for reliable comparisons across health systems despite differences in their local EMR structures.

TriNetX employs automated quality control procedures to detect and correct errors, including missing values, implausible laboratory results, and duplicate records. Data are continuously monitored for consistency, and member institutions are required to validate their data pipelines. The reliability of the TriNetX platform has been demonstrated in prior validation studies, including replication of results from randomized controlled trials [11,12,13]. A detailed description of the TriNetX data model and validation framework was published by Palchuk et al. [13]. Additionally, Mankowski et al. recently demonstrated the validity of a similar EMR-derived platform (Epic Cosmos) for kidney transplant research, with results comparable to the Scientific Registry of Transplant Recipients (SRTR) [14]. In recent years, TriNetX has been adopted in transplant research, with several studies published between 2022 and 2025 demonstrating its utility in investigating clinical variables typically unavailable in national databases, such as the SRTR, including the effects of medications like sodium-glucose cotransporter-2 inhibitors (SGLT2i), infections, and post-transplant malignancies [15,16,17,18].

This study is a secondary analysis of existing, de-identified data and does not involve direct interaction with or intervention on human subjects; therefore, it is exempt from informed consent. The data are de-identified in accordance with Section §164.514(a) of the HIPAA Privacy Rule, with the de-identification process formally attested to by a qualified expert as defined in Section §164.514(b) (1). TriNetX confirmed this formal determination in December 2020 [19].

This study was designed and reported in accordance with the Strengthening the Reporting of Observational Studies in Epidemiology (STROBE) guidelines [20]. The complete STROBE checklist can be found in the Supplementary Table S1.

### 2.2. Study Design and Population

The TriNetX database was used to conduct a retrospective, propensity-score-matched case–control study evaluating 3-, 6-, and 12-month graft and infectious outcomes in SLK recipients. Patients were included if they underwent a liver transplant between 31 December 2000, and 31 December 2020, with a kidney transplant performed within one day before or after the liver transplant. In TriNetX, outcome timing is based on the “index event,” defined as the day of the liver transplant procedure. Patients were excluded if they had received another solid organ transplant (intestine, pancreas, heart, or lung) or a bone marrow transplant before or after the SLK. Recipients with a prior liver or kidney transplant were not excluded, as liver transplant failure or re-transplantation was an outcome of interest; instead, cohorts were matched to ensure a similar proportion of re-transplantation in each group.

**Figure 1:**
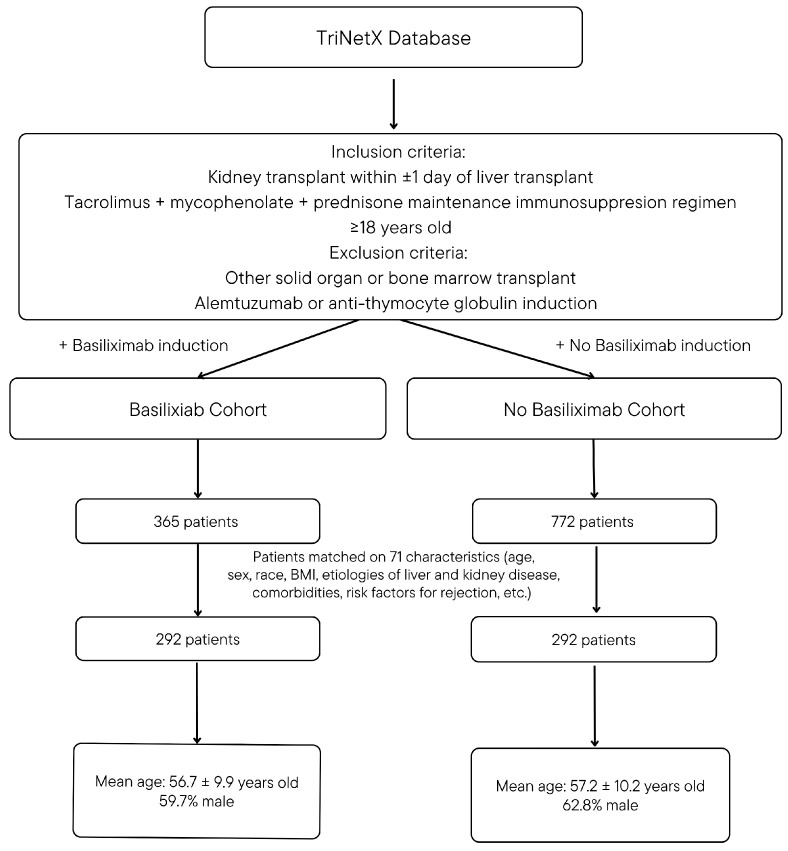
Cohort design.

After identifying first-time SLK recipients, cohorts were limited to patients receiving maintenance immunosuppression with either tacrolimus (TAC) plus mycophenolate mofetil (MMF) or mycophenolic acid (MPA) plus prednisone. The basiliximab (Bas) cohort included patients who received basiliximab induction, as indicated by the corresponding RxNorm code, while the no basiliximab (No Bas) cohort excluded any patients who received basiliximab. Patients with RxNorm codes for other induction agents, including anti-thymocyte globulin or alemtuzumab, were excluded. Steroid induction was permitted in either cohort. Using this methodology, 365 patients were identified in the Bas cohort, and 772 patients were identified in the No Bas cohort. Figure 1 includes an overview of methodology and cohort design. 

### 2.3. Propensity Score Matching Variables

Cohorts were matched on 71 selected characteristics (codes) identified in the EMR from 10 years to 1 day prior to the liver transplant procedure. Recipients were matched on demographics, including age at the time of the transplant procedure (age at index), sex, and documented race/ethnicity (Black or African American, Hispanic or Latino, Not Hispanic or Latino, White, American Indian or Alaska Native, or Asian). Recipients were also matched on established risk factors for rejection, including diffuse mesangial proliferative glomerulonephritis, focal segmental glomerulosclerosis (FSGS), systemic lupus erythematosus (SLE), diabetes mellitus, Human immunodeficiency virus (HIV), body mass index (BMI), and overweight/obesity status [21,22,23]. Prior liver or kidney transplant history, including previous transplant procedures, rejection episodes, or transplant failure diagnoses, was also matched. Donor type (deceased, living, or deceased or living) was included in the matching.

Etiologies of liver and kidney disease were matched, including viral hepatitis, alcoholic liver disease, fatty liver, nonalcoholic steatohepatitis, liver cell carcinoma, primary biliary cirrhosis, primary sclerosing cholangitis, autoimmune hepatitis, essential hypertension, and hepatorenal syndrome. To account for severity of illness prior to transplantation, recipients were matched on clinical proxies including portal hypertension, abdominal paracentesis, hepatic encephalopathy, respiratory failure, shock, dialysis modalities (hemodialysis, peritoneal dialysis, hemofiltration, or other continuous renal replacement therapies), critical care services, MELD score, and vasopressor or supportive therapies (midodrine, vasopressin, norepinephrine, phenylephrine, albumin, vitamin K, octreotide).

Immunologic risk and sensitization were addressed by matching on calculated panel reactive antibody, HLA antibodies (of various types and assays), plasmapheresis, prior transfusions, and previous pregnancy history [24]. For recipients without a recorded MELD score, laboratory values, including sodium, total bilirubin, platelets, INR, and creatinine, were used as surrogates. Additional labs used in matching included albumin, cytomegalovirus IgG, Epstein–Barr virus capsid IgG, and varicella zoster virus IgG. TriNetX provides limited donor characteristic data. A complete list of specific codes used for matching is included in Supplementary File S1.

### 2.4. Primary Analysis: Graft and Infectious Outcomes

The primary objective of this analysis was to evaluate graft and infectious outcomes in SLK recipients at 14–90, 14–180, and 14–365 days post-transplant. “Liver or kidney transplant rejection” was defined as a diagnosis of rejection or treatment for rejection, including anti-thymocyte globulin, IVIG, or methylprednisolone (≥500 mg). The analysis began 14 days after transplantation to account for methylprednisolone used as induction therapy and to ensure that treated or diagnosed rejection events were captured. Liver and kidney rejection outcomes were defined separately based on the corresponding diagnosis codes.

Because TriNetX relies on EMR codes and does not specify whether rejection was biopsy-proven, liver and kidney biopsy procedure codes were also analyzed as separate outcomes to indicate the proportion of recipients who underwent biopsies. Kidney allograft failure was defined as an eGFR ≤ 15 mL/min/1.73 m^2^ (CKD-EPI). Liver primary non-function was defined as the need for liver re-transplantation within 7 days post-transplant [25]. Delayed kidney graft function was defined using the widely accepted definition of requiring dialysis within the first week post-transplant and was further differentiated into “dialysis” versus “hemodialysis only” [26]. “Dialysis” included hemodialysis, peritoneal dialysis, continuous renal replacement therapy, or hemofiltration, while “hemodialysis” included only hemodialysis procedures. The evaluation period for liver allograft failure and delayed kidney graft function was 2–8 days after the index event, defined as the liver transplant procedure, with the kidney transplant occurring within one day before or after the liver transplant. The 2-day start period was chosen to avoid capturing dialysis that may have occurred one day post-liver transplant but before the kidney transplant.

Mortality was defined using the “deceased” status in TriNetX demographic data. Hospitalizations were assessed as the mean number of inpatient days, based on hospital inpatient or observation care service codes.

Infectious outcomes were identified at 3-, 6-, and 12-month post-transplant. Opportunistic viral outcomes included both diagnosed and/or viremic episodes, defined as ≥1000 units/mL or ≥3 log copies/mL, as per LOINC. Opportunistic viral infections included CMV, VZV, EBV, JC virus, and BK virus, as well as a composite of the five viral outcome codes (See Supplementary File S1). Sepsis was defined using ICD-10-CM codes for sepsis. We looked at viral, bacterial, or other infectious pneumonia using the ICD-10-CM codes for pneumonia, unspecified organism, viral pneumonia, and bacterial pneumonia. Lastly, we looked at pyelonephritis using the “acute pyelonephritis” and “kidney transplant infection” ICD-10-CM codes.

### 2.5. Secondary Analysis: Descriptive Outcomes

The mean eGFR, as calculated by the creatinine-based formula (CKD-EPI), is reported as the most recent measurement within the defined post-transplant period (14 days, 3 months, 6 months, and 1 year). Liver function tests (LFTs) included AST, ALT, INR, and Total bilirubin, and are reported as the mean value, using the most recent occurrence (values) available. Tacrolimus trough levels (mass/volume) are also reported at 14 days, 3 months, 6 months, and 1-year post-transplant.

### 2.6. Statistical Analysis

Balanced cohorts were generated using TriNetX’s built-in propensity score matching method with a greedy nearest-neighbor approach to reduce confounding in this observational study. Propensity scores were calculated using logistic regression based on user-selected baseline variables, and the two cohorts were matched 1:1 using greedy nearest-neighbor matching, in which each patient in the treatment group (Bas) is paired with the control patient (No Bas) with the closest propensity score. Covariates were considered adequately matched if post-matching comparisons yielded *p*-values ≥ 0.05.

Time-to-event outcomes, including rejection, graft failure, graft biopsy, dialysis, mortality, liver re-transplantation, delayed graft function (DGF), hospitalizations, cytomegalovirus (CMV) viremia, Epstein–Barr virus (EBV) viremia, varicella zoster virus (VZV) viremia, BK viremia, JC viremia, composite viremia, sepsis, pneumonia, and pyelonephritis, were analyzed using Kaplan–Meier methods. Differences between groups were assessed using the log-rank test, and hazard ratios with proportionality testing were reported. Statistical significance was defined as a *p*-value of ≤0.05. Quantitative variables were analyzed as mean ± standard deviation (SD), and differences in these values were analyzed using independent *t*-tests, with significance set at *p* ≤ 0.05. For propensity score matching, all continuous quantitative variables were included as continuous covariates without transformation. Missing data were handled per TriNetX default procedures, which exclude patients without available data for a given variable from that variable’s analysis but retain them for other analyses.

## 3. Results

### 3.1. Propensity Score Matching Results

There were 292 patients in each cohort after matching. Cohorts were well-matched in terms of demographics, including age at the time of transplant, sex, and reported race/ethnicity. There were no significant differences in pre-transplant diagnoses, etiologies of kidney or liver disease, or comorbid conditions. In addition, the cohorts were well-balanced in terms of complications of cirrhosis, critically ill status, and specified laboratory values. Risk factors for sensitization and immunologic risk were well-matched between cohorts. Graft type, living, deceased, or either, was well-matched between cohorts. Patients who had received a prior liver or kidney transplant were evenly matched between the cohorts, defined by prior liver or kidney transplant procedure, rejection, or graft failure diagnosis. cPRA values were not significantly different between cohorts (*p* > 0.05). Table 1 includes a complete list of post-matching characteristics in the Bas and No Bas cohorts.

Propensity score matching results with *n* = 10 were excluded from the table, as TriNetX does not report values below 10 to ensure patient privacy and de-identification. After matching, the following results had less than 10 samples and were evenly matched in each cohort (*p* > 0.05): Asian, American Indian or Alaskan, Human immuno-deficiency virus (HIV), Primary sclerosing cholangitis, Autoimmune hepatitis, Systemic lupus erythematosus (SLE), Chronic nephritic syndrome with diffuse mesangial proliferative glomerulonephritis, Nephritic syndrome with focal and segmental glomerular lesions, pregnancy, plasmapheresis, and prior kidney transplant. A table with the pre-propensity score matching results and results with *n* = 10 can be found in Supplementary Table S2.

### 3.2. Delayed Kidney Graft Function/Liver Primary Non-Function

There was no significant difference in delayed kidney graft function, defined as the need for dialysis within 7 days post-transplant. In the Bas cohort, 27.45% required dialysis (hemodialysis, peritoneal dialysis, CRRT, or hemofiltration) compared with 25.54% in the No Bas cohort. When limited to hemodialysis specifically, 12.94% of the Bas cohort and 18.08% of the No Bas cohort underwent the procedure within 7 days post-transplant.

Liver primary non-function, defined as the need for liver re-transplantation within 7 days, occurred more frequently in the Bas cohort (2.75% vs. 0.39%, *p* = 0.04). See Table 2 for complete results.

### 3.3. Graft and Recipient Outcomes

**Figure 2:**
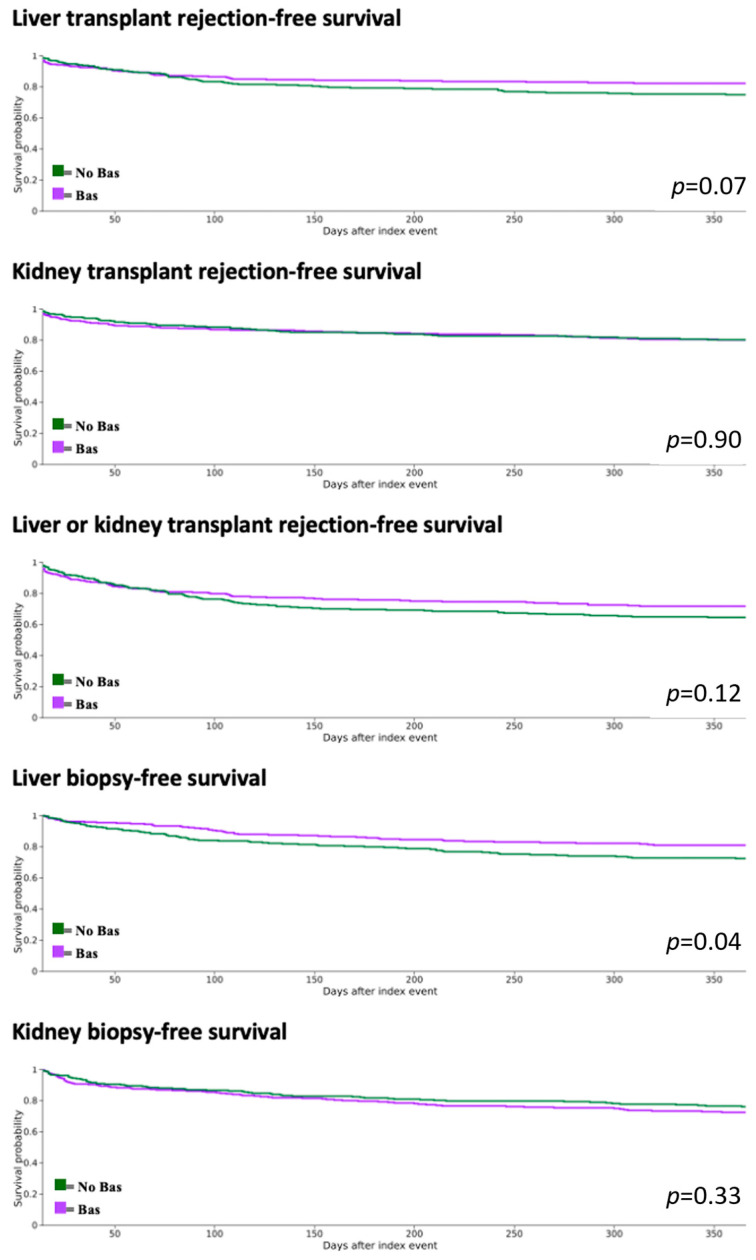
Kaplan–Meier Survival Curves for graft rejection and biopsy, with *p*-value demonstrated for each curve. Curves represent time-to-event analyses depicting the proportion of patients remaining event-free over time.

Kaplan–Meier analysis demonstrated similar incidence of kidney transplant rejection, liver transplant rejection, composite rejection, kidney graft failure, hemodialysis, liver re-transplantation, hospitalizations, and mortality between the cohorts. Rates of liver biopsy were higher in the No Bas cohort at 3, 6, and 12 months post-transplant, whereas kidney biopsy rates were comparable between groups. The results are presented in Table 3 below. Kaplan–Meier Survival curves are shown in Figure 2 for liver and kidney transplant rejection, combined rejection, as well as liver and kidney biopsy-free survival.

### 3.4. Infectious Outcomes

The No Bas cohort had higher rates of CMV at all time points (30.47% vs. 22.97%, *p* = 0.03 at 1 year) and EBV at 6 months and 1 year (2.60% vs. 0.36%, *p* = 0.03 at 1 year), although the incidence of EBV was low. Composite viremia (CMV, EBV, BK, JC, and VZV) was higher in the No bas Cohort at all time points (35.49% vs. 27.00%, *p* = 0.01). There was no significant difference in the rates of pneumonia, pyelonephritis, or sepsis between the two cohorts. The full results are presented in Table 4.

### 3.5. Descriptive Outcomes

**Figure 3:**
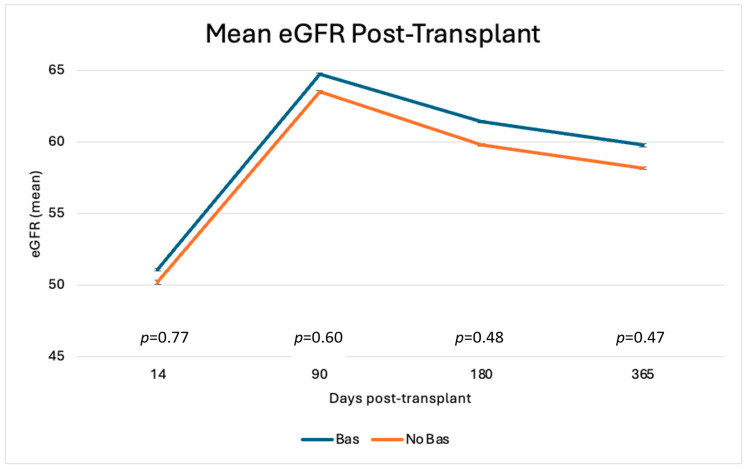
Mean eGFR (mL/min/1.73 m^2^) ± standard error (SE) at 14 days (2–14 days), 3 months (14–90 days), 6 months (90–180 days), and 1 year (180–365 days.) *p*-values from two-sample *t*-tests comparing the groups at each time point are displayed on the graph.

**Figure 4:**
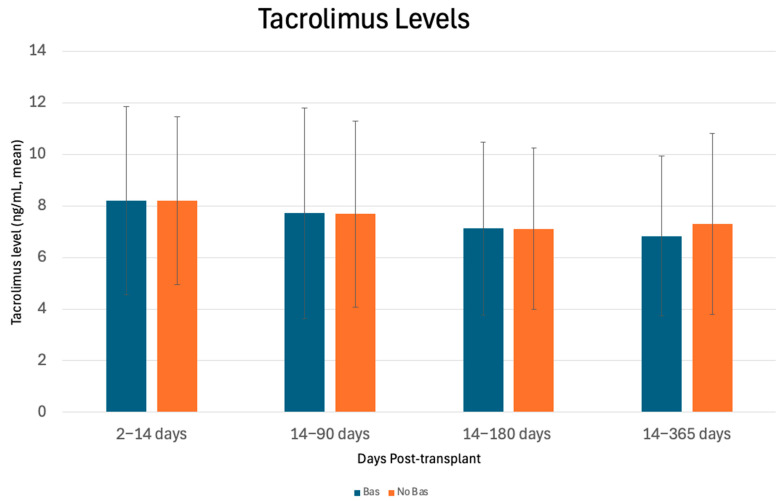
Mean Tacrolimus levels (mass/volume) ± standard deviation (SD) at 14 days (2–14 days), 3 months (14–90 days), 6 months (90–180 days), and 1 year (180–365 days) post-transplant.

There were no significant differences in mean eGFR or LFTs between cohorts at 14 days, 3 months, 6 months, or 1 year post-transplant, as shown in Figure 3 and Table 5, respectively. The mean tacrolimus levels were similar at 14 days, 3 months, 6 months, and 1 year as shown in Figure 4. When evaluating the immediate post-transplant period, tacrolimus trough levels were lower in the Bas cohort at day 4 (5.5 ng/mL vs. 7.8 ng/mL, *p* = 0.002), suggesting lower dosages or delayed introduction of tacrolimus with basiliximab.

## 4. Discussion

In this multicenter retrospective analysis, we found that basiliximab induction did not significantly alter rates of kidney or liver transplant rejection. Our findings are consistent with prior studies that have evaluated induction strategies in SLK recipients. Ruiz et al. compared basiliximab with corticosteroid-only induction and reported no significant difference in liver or kidney biopsy-proven acute rejection at 1 year [9]. However, their observed kidney transplant rejection rates were lower than in our cohort, with 12-month composite liver or kidney transplant rejection occurring in 15% of corticosteroid-only and 21% of basiliximab recipients, compared to 28% and 35%, respectively, in our study. Kidney rejection at 12 months was lower in the Ruiz et al. cohort (5% corticosteroids, 0% basiliximab) compared to our findings (19% in both groups). By contrast, liver rejection rates at 12 months were comparable between studies (Ruiz et al.: 12% with corticosteroids vs. 21% with basiliximab; our study: 17% with basiliximab vs. 25% without basiliximab).

Another multicenter study evaluating different induction agents in SLK recipients found no added benefit of IL-2 receptor antagonist induction agents, such as basiliximab, in lowering 1-year liver or kidney transplant rejection when compared to no induction [10]. This study by Abdul et al. also reported lower rejection rates overall than our study, with 12-month kidney rejection occurring in 6.2% of recipients without induction therapy, compared to 6.6% with IL-2 receptor antagonist therapy, and liver rejection in 9.0% versus 9.7%, respectively. These differences in rejection incidence may be attributable to methodological variation: both prior studies assessed only biopsy-proven acute rejection, whereas our analysis used a composite endpoint including ICD-10 diagnosis codes and treatment for rejection, which may capture clinically suspected and empirically treated cases not confirmed by biopsy.

Our findings also align with a large national OPTN analysis, which examined outcomes in SLK recipients between 1996 and 2016 by induction regimen [4]. In an unadjusted Kaplan–Meier analysis, patient survival at 5 years appeared superior with IL-2RA (74%) compared to T-cell depletion (68%) and no induction (71%). However, after multivariable adjustment, induction type was not associated with differences in either patient or graft survival. These results suggest that the induction strategy may not impact long-term survival outcomes in SLK recipients; however, they did not examine earlier outcomes, such as acute rejection or infection risks. Currently, there is limited data available comparing the impact of induction agents on patient and graft outcomes, with the three mentioned being the only ones identified [4,9,10].

Delayed kidney graft function was similar between groups in our study (27% in the basiliximab group vs. 25% in the no-basiliximab group), aligning with the rates reported by Ruiz et al. (27% in the corticosteroids group vs. 26% in the basiliximab group) [9]. The incidence of primary liver non-function was higher in the basiliximab cohort, though the absolute numbers were low. While this difference reached statistical significance (2.75% vs. 0.39%, *p* = 0.04), the low overall frequency of events warrants cautious interpretation.

Interestingly, rates of liver biopsy were higher in the No Bas cohort, yet this did not correspond to a higher incidence of diagnosed or treated rejection. This discrepancy may reflect greater clinical caution or a lower threshold to pursue biopsy in the absence of basiliximab induction, or it could be related to limitations in EMR coding, which may underreport actual rejection episodes. There was no observed difference in liver function tests at 3, 6, or 12 months post-transplant.

At post-transplant day 4, tacrolimus trough levels were lower in the Bas cohort (5.5 vs. 7.8, *p* = 0.002), suggesting that delayed initiation or lower dosages of tacrolimus were initially used in this cohort. While basiliximab is sometimes used in liver-only transplantation to delay the initiation of renal-toxic calcineurin inhibitors and has been associated with less kidney injury, no differences were observed in delayed kidney graft function, the need for hemodialysis, or mean eGFR at up to 1 year post-transplant, suggesting that overall kidney graft performance was comparable between the cohorts [27].

We also observed higher rates of CMV and composite viral infections in the No Bas cohort at all time points, as well as higher rates of EBV viremia at 6 and 12 months. These findings align with the study by Ruiz et al. [9], which reported higher overall infection rates at 3 months post-transplant, defined by positive blood or body fluid cultures. In our study, no differences were seen in bacterial complications such as pneumonia, pyelonephritis, or sepsis. The higher incidence of viral infections in the No Bas cohort may be related to greater exposure to corticosteroids, which have been well established to increase susceptibility to infection, especially CMV viral reactivation and replication [28].

Our study adds to the limited body of evidence on the role of induction therapy in SLK transplantation and has several implications for clinical practice. The lack of difference in rejection or graft outcomes between the basiliximab and no-induction (apart from corticosteroids) cohorts suggests that routine IL-2 receptor antagonist induction may not be necessary for most SLK recipients, who benefit from the immunoprotective effect of the liver graft. Interestingly, we observed a higher rate of liver biopsy in the no-basiliximab cohort, without a corresponding increase in diagnosed or treated rejection. This may reflect greater clinical suspicion or a lower biopsy threshold for patients not receiving induction therapy, rather than a true difference in rejection risk. In clinical practice, these findings may support a more selective use of basiliximab, reserved for SLK recipients with high immunologic risk, impaired kidney function, or contraindications to the early use of calcineurin inhibitors, while avoiding unnecessary induction in standard-risk patients. Furthermore, the higher rate of viral infections observed in the no-basiliximab group may reflect higher corticosteroid exposure in this cohort, reinforcing the importance of minimizing steroid use where possible. Taken together, these findings suggest that an induction approach relying solely on corticosteroids may be both safe and practical for most SLK recipients; however, further robust prospective studies are needed to clarify this strategy and identify which subgroups may benefit from induction with basiliximab.

### Limitations/Generalizability

Our study adds to the scant literature available on the impact of induction agents on patient, graft, and infectious outcomes in SLK transplantation. Limitations to this study include its retrospective design and the dependence on accurate EMR coding by healthcare providers. We attempt to limit our reliance on the accurate coding of ICD diagnoses by utilizing lab results, such as for the definition of viremia, and medication administration, including high-dose methylprednisone, anti-thymocyte globulin, or IVIG to capture rejection episodes. By including a comprehensive list of codes for each result, we attempt to limit the nuances in EMR coding practices. TriNetX also does not provide donor-derived data, thereby limiting the consideration of donor-related factors that may impact outcomes.

Another limitation to this study is that rejection was defined as a diagnosis of, using ICD-10 codes, or treatment for rejection, rather than biopsy-proven rejection, as TriNetX is unable to provide information on which rejections were biopsy-proven. We attempted to mitigate this limitation by also providing separate results for liver and kidney biopsies to determine which patients received these procedures. Nonetheless, this definition of rejection may under- or over-capture true rejection episodes. Additionally, data on cPRA was not available for all patients in the cohorts. To further match the cohorts on immunologic risk, we matched on previously described risk factors for rejection, including FSGS, diffuse mesangial proliferative glomerulonephritis, SLE, diabetes mellitus, HIV, BMI, and overweight/obesity status. We also matched on HLA antibodies, the need for plasmapheresis, prior transfusions, or prior pregnancies that may increase the risk of sensitization in patients.

This study utilized the TriNetX Research Network, which aggregates de-identified electronic health record data from multiple healthcare organizations across the United States, enhancing the external validity of our findings. The large, diverse, and geographically distributed cohort increases the generalizability of the results to the broader population of adult SLK transplant recipients in the U.S. However, potential limitations to generalizability include variability in center-specific practices, incomplete details of precise immunosuppression dosages, and possible differences in post-transplant management that may not be fully represented in the database. Therefore, while the multicenter design of this study supports generalizability, the results should be interpreted in the context of these inherent limitations of real-world data.

## 5. Conclusions

SLK recipients who did not receive basiliximab induction had similar graft outcomes to those who did, although higher rates of liver biopsy and early viral infections were observed in the no-basiliximab cohort. The higher liver biopsy rate may reflect greater clinical suspicion among clinicians when induction was not used, rather than a true difference in rejection risk, as it did not translate into increased diagnosis of or treatment for rejection. Overall, our findings suggest that basiliximab induction may not be routinely necessary for most SLK recipients and that a more selective, risk-based approach is appropriate.

## Figures and Tables

**Table 1:** Propensity Score Matching Results. This table summarizes the characteristics of the basiliximab (Bas) and no basiliximab (No Bas) cohorts following propensity score matching. Continuous variables (e.g., age, BMI, laboratory values) are presented as mean ± standard deviation (SD), while categorical variables (e.g., gender, race/ethnicity, and diagnoses) are reported as the number of individuals (*n*) with the corresponding percentage of the cohort (%).

	Cohort Characteristics, After Propensity-Score Matching (Mean ± SD; *n* (% Cohort))		
	Bas	No Bas	*p* Value
Demographics			
Age	56.7 ± 9.9	57.2 ± 10.2	0.62
Male	174 (59.7%)	183 (62.8%)	0.44
Black or African American	52 (17.7%)	43 (14.6%)	0.31
Hispanic or Latino	17 (5.9%)	29 (10.1%)	0.07
White	193 (66.3%)	200 (68.4%)	0.60
Diagnoses			
BMI	28.4 ± 6.3	28.0 ± 5.9	0.54
Overweight and obesity	105 (35.8%)	109 (37.2%)	0.73
Viral hepatitis	91 (31.3%)	88 (30.2%)	0.79
Unspecified viral hepatitis C	70 (24.0%)	68 (23.3%)	0.84
Unspecified viral hepatitis B	14 (4.9%)	10 (3.5%)	0.40
Alcoholic liver disease	148 (50.7%)	146 (50.0%)	0.87
Fatty liver	67 (22.9%)	75 (25.7%)	0.44
Nonalcoholic steatohepatitis	103 (35.4%)	89 (30.6%)	0.22
Liver cell carcinoma	39 (13.2%)	37 (12.8%)	0.90
Primary biliary cirrhosis	15 (5.2%)	13 (4.5%)	0.70
Primary hypertension	187 (63.9%)	187 (64.2%)	0.93
Diabetes mellitus	148 (50.7%)	149 (51.0%)	0.93
Hepatorenal syndrome	148 (50.7%)	146 (50.0%)	0.87
Markers of Illness Severity			
Portal hypertension	225 (77.1%)	217 (74.3%)	0.44
Abdominal paracentesis	137 (46.9%)	143 (49.0%)	0.62
Hepatic encephalopathy	78 (26.7%)	73 (25.0%)	0.63
Respiratory failure	97 (33.3%)	97 (33.7%)	0.93
Shock	64 (21.9%)	70 (24.0%)	0.55
Hemodialysis	110 (37.8%)	119 (40.6%)	0.50
Peritoneal dialysis, CRRT, hemofiltration	63 (21.5%)	53 (18.1%)	0.30
Critical Care services	110 (37.8%)	114 (38.9%)	0.80
Model for end-stage liver disease score (*n*, % with value)	37.9 ± 8.2 (17, 5.9%)	36.4 ± 8.0 (10, 3.5%)	0.65
Medications			
Midodrine	146 (50.0%)	149 (51.0%)	0.80
Vasopressin	48 (16.3%)	54 (18.4%)	0.51
Norepinephrine	61 (20.8%)	65 (22.2%)	0.69
Phenylephrine	77 (26.4%)	73 (25.0%)	0.70
Albumin	198 (67.7%)	208 (71.2%)	0.37
Vitamin K	96 (33.0%)	97 (31.3%)	0.66
Octreotide	116 (39.9%)	118 (40.3%)	0.93
Labs			
Sodium [moles/volume]	135.5 ± 5.0	135.3 ± 4.8	0.59
Bilirubin, total [mass/volume]	5.0 ± 8.2	6.1 ± 10.1	0.20
Platelets [#/volume]	90.4 ± 66.4	92.3 ± 66.5	0.74
INR in Plasma or Blood	1.6 ± 0.6	1.6 ± 0.7	0.09
Albumin [mass/volume]	3.1 ± 0.7	3.1 ± 0.7	0.85
Creatinine [mass/volume]	4.0 ± 2.9	3.7 ± 2.6	0.25
Cytomegalovirus IgG Ab [units/volume] in Serum or Plasma (*n*, % cohort)	5.4 ± 12.3 (21, 7.3%)	26.5 ± 79.1 (27, 9.4%)	0.23
Varicella zoster virus IgG Ab [Presence] in Serum	10 (3.5%)	21 (7.3%)	0.50
Epstein–Barr virus capsid IgG Ab [Presence] in Serum	10 (3.5%)	14 (4.9%)	0.40
HLA Ab in Serum by Flow cytometry (FC) (*n*, % with value)	0.4 ± 0.5 (17, 5.9%)	0.3 ± 0.5 (10, 3.5%)	0.26
Procedures			
Backbench preparation of living donor renal allograft	11 (3.91%)	11 (3.91%)	1
Backbench preparation of deceased donor renal allograft	217 (74.22%)	217 (80.01%)	0.11
Backbench preparation of deceased whole liver graft	217 (74.44%)	243 (83.20%)	0.06
Backbench preparation of deceased or living renal allograft	38 (12.89%)	56 (19.14%)	0.05
Transfusion of Red Blood Cells	58 (19.8%)	43 (14.6%)	0.20
Transfusion, blood or components	44 (14.9%)	44 (14.9%)	1
Previous liver or kidney transplant			
Liver transplant	11 (3.8%)	11 (3.8%)	1
Kidney transplant rejection diagnosis	14 (4.9%)	12 (4.1%)	0.68
Kidney transplant failure diagnosis	16 (5.6%)	15 (5.3%)	0.85
Liver transplant rejection diagnosis	13 (4.5%)	13 (4.5%)	1
Liver transplant failure diagnosis	14 (4.9%)	14 (4.5%)	0.84

Abbreviations: Bas = Basiliximab cohort, No Bas = No Basiliximab cohort, SD = standard deviation, BMI = body mass index, CRRT = continuous renal replacement therapy, # = number, HLA= Human Leukocyte Antigen.

**Table 2:** Cumulative incidence and hazard ratios with 95% confidence intervals of delayed kidney graft function and primary liver non-function in each cohort. Delayed kidney graft function is further subdivided into need for any dialysis (hemodialysis, peritoneal dialysis, continuous renal replacement therapy, or hemofiltration) versus hemodialysis only.

		Cumulative Incidence (%)	*p*	Hazard Ratio (95% Confidence Interval)
Delayed graft function (need for any dialysis)	Bas	27.45%	0.64	1.081 (0.771, 1.515)
	No Bas	25.54%		
Delayed graft function (need for hemodialysis)	Bas	12.94%	0.08	0.677 (0.433, 1.059)
	No Bas	18.08%		
Primary liver non-function	Bas	2.75%	0.04	7.038 (0.866, 57.207)
	No Bas	0.39%		

Abbreviations: Bas = Basiliximab cohort, No Bas = No Basiliximab cohort.

**Table 3:** Graft and patient outcomes in each cohort at 3, 6, and 12 months post-transplant. Reported as cumulative incidence (CI) in % for categorical variables and as the mean number (#) for hospitalizations.

Outcome	Cohort	3-Month CI (%)/Mean #	*p*	6-Month CI (%)/Mean #	*p*	1 Year CI (%)/Mean #	*p*
Kidney transplant rejection (Diagnosis only)	Bas	10.41%	0.88	15.35%	0.93	19.97%	0.90
	No bas	10.19%		15.30%		19.79%	
Liver transplant rejection (Diagnosis only)	Bas	12.60%	0.97	16.14%	0.15	17.80%	0.07
	No bas	12.81%		21.82%		25.08%	
Kidney or liver transplant rejection (Diagnosis or treated)	Bas	17.25%	0.48	24.16%	0.11	28.29%	0.12
	No bas	20.08%		31.42%		35.47%	
Liver biopsy	Bas	7.61%	0.004	14.55%	0.001	18.16%	0.04
	No bas	15.39%		25.30%		25.47%	
Kidney biopsy	Bas	12.90%	0.95	20.74%	0.68	27.55%	0.33
	No bas	12.73%		21.87%		23.93%	
Hemodialysis	Bas	15.72%	0.82	17.20%	0.90	18.01%	0.93
	No bas	16.44%		17.53%		17.54%	
Kidney graft failure (eGFR < 15)	Bas	12.91%	0.30	14.03%	0.15	15.28%	0.05
	No bas	16.08%		18.71%		22.36%	
Liver re-transplant	Bas	0.38%	1.00	0.38%	0.78	0.38%	0.78
	No bas	0.38%		0.38%		0.38%	
Hospitalizations (mean #)	Bas	19.83	0.15	25.55	0.17	29.65	0.25
	No bas	17.06		21.73		25.56	
Mortality	Bas	2.46%	0.61	3.93%	0.24	7.83%	0.98
	No bas	3.17%		6.17%		7.64%	

Abbreviations: CI = Cumulative incidence, Bas = Basiliximab cohort, No bas = No Basiliximab cohort.

**Table 4:** Incidence of viral and infectious outcomes in each cohort at 3, 6, and 12 months post-transplant reported as the cumulative incidence (CI) with corresponding *p* values reported.

Outcome	Cohort	3-Month CI (%)	*p*	6-Month CI (%)	*p*	1 Year CI (%)	*p*
CMV	Bas	5.25%	0.003	14.57%	0.02	22.97%	0.03
	No bas	12.38%		21.93%		30.47%	
EBV	Bas	0.35%	0.17	0.35%	0.06	0.36%	0.03
	No bas	1.42%		2.18%		2.60%	
BK Virus	Bas	1.79%	0.54	5.12%	0.4	6.35%	0.81
	No bas	2.51%		6.63%		6.63%	
JC Virus	Bas	0.00%	1	0.00%	1	0.00%	0.31
	No bas	0.00%		0.38%		0.38%	
VZV	Bas	0.00%	0.31	1.12%	0.70	1.98%	0.50
	No bas	0.35%		1.47%		2.72%	
Composite Viremia (CMV, EBV, BK, JC, VZV)	Bas	7.04%	0.002	18.60%	0.006	27.00%	0.01
	No bas	15.22%		27.83%		35.49%	
Composite pneumonia	Bas	10.58%	0.57	16.58%	0.61	20.60%	0.09
	No bas	9.17%		11.54%		15.15%	
Pyelonephritis	Bas	5.01%	0.47	7.94%	0.64	12.04%	0.17
	No bas	6.52%		6.96%		8.20%	
Sepsis	Bas	15.37%	0.99	22.15%	0.85	28.51%	0.81
	No bas	15.47%		22.93%		27.27%	

Abbreviations: CI = Cumulative incidence, Bas = Basiliximab cohort, No bas = No Basiliximab cohort.

**Table 5:** Liver function tests (mean values ± standard deviation (SD)) at 14 days (1–14 days), 3 months (14–90 days), 6 months (14–180 days), and 12 months (14–365 days) post-transplant.

Outcome	Cohort	14 Days (Mean ± SD)	*p*	3 Months (Mean ± SD)	*p*	6 Months (Mean ± SD)	*p*	12 Months (Mean ± SD)	*p*
AST	Bas	30.99 ± 45.84	0.24	30.65 ± 67.93	0.66	60.60 ± 459.87	0.56	61.43 ± 429.12	0.41
	No bas	52.31 ± 271.39		34.36 ± 103.50		90.95 ± 679.26		37.51 ± 101.38	
ALT	Bas	51.77 ± 66.3	0.22	31.47 ± 46.91	0.44	44.7 ± 199.66	0.63	42.88 ± 184.96	0.51
	No bas	64.14 ± 136.65		35.57 ± 63		54.56 ± 224.97		34.54 ± 51.34	
Total bilirubin	Bas	1.71 ± 3.17	0.42	0.82 ± 2.79	0.58	0.95 ± 2.96	0.13	0.93 ± 2.78	0.19
	No bas	1.97 ± 3.52		0.95 ± 1.87		1.5 ± 4.24		1.35 ± 4.07	
INR	Bas	1.16 ± 0.30	0.31	1.17 ± 0.35	0.36	1.24 ± 0.61	0.40	1.17 ± 0.48	0.86
	No bas	1.19 ± 0.36		1.21 ± 0.46		1.19 ± 0.49		1.18 ± 0.41	

## Data Availability

The data presented in this study are available in TriNetX: https://live.trinetx.com/ (accessed on 20 August 2025). Users must request access to this database through their institution; the data is not openly available.

## Supplementary Materials

The following supporting information can be downloaded at: https://www.mdpi.com/article/10.3390/antib14040091/s1, Table S1: STROBE Statement—Checklist of items that should be included in reports of cohort studies, Table S2: Propensity Score Matching Results. File S1: Methods: Definition of Cohorts, 1:1 Propensity Score-Matching Criteria, and Outcomes Definitions.

## References

[1] Liver-Scientific Registry of Transplant Recipients. https://srtr.transplant.hrsa.gov/ADR/Chapter?name=Liver&year=2022.

[2] Parajuli S., Hidalgo L.G., Foley D. (2022). Immunology of simultaneous liver and kidney transplants with identification and prevention of rejection. Front. Transpl..

[3] Kim J.-Y., Bin Kim H., Kim J.-M., Kwon H.E., Kim Y.H., Ko Y., Sung F.S., Jung J.H., Baek C.H., Kim H. (2024). Immunoprotective Effect of Liver Allograft on Patients with Combined Liver and Kidney Transplantation. Ann. Transpl..

[4] Kamal L., Yu J.W., Reichman T.W., Kang L., Bandyopadhyay D., Kumar D., King A., Gautam U., Bhati C., Yakubu I. (2020). Impact of Induction Immunosuppression Strategies in Simultaneous Liver/Kidney Transplantation. Transplantation.

[5] Boyd A., Brown A., Patel J., Nightingale P., Perera M.T.P., Ferguson J., Neuberger J., Rajoriya N. (2021). Basiliximab with Delayed Tacrolimus Improves Short-Term Renal Outcomes Post-Liver Transplantation—A Real-World Experience. Transplant. Proc..

[6] Ali H., Mohammed M., Fülöp T., Malik S. (2023). Outcomes of thymoglobulin versus basiliximab induction therapies in living donor kidney transplant recipients with mild to moderate immunological risk–a retrospective analysis of UNOS database. Ann. Med..

[7] Yao X., Weng G., Wei J., Gao W. (2016). Basiliximab induction in kidney transplantation with donation after cardiac death donors. Exp. Ther. Med..

[8] Basiliximab Induction Therapy for Live Donor Kidney Transplantation: A Long-Term Follow-Up of Prospective Randomized Controlled Study-PubMed. https://pubmed-ncbi-nlm-nih-gov.libux.utmb.edu/18327678/.

[9] Ruiz I., Sparkes T., Masters B., Barth R., Maluf D., Freedman S. (2022). Impact of Steroid Only Induction on Rejection in Simultaneous Liver-Kidney Transplantation. Prog. Transplant..

[10] AbdulRahim N., Anderson L., Kotla S., Liu H., Ariyamuthu V.K., Ghanta M., MacConmara M., Tujios S.R., Mufti A., Mohan S. (2019). Lack of Benefit and Potential Harm of Induction Therapy in Simultaneous Liver-Kidney Transplants. Liver Transplant..

[11] TriNetX Admin TriNetX Real-World Evidence Platform Validates Outcomes of Randomized Clinical Trials. https://trinetx.com/press-releases/real-world-evidence-platform-validates-outcomes-of-randomized-clinical-trials/.

[12] Stapff M.P. (2018). Using real world data to assess cardiovascular outcomes of two antidiabetic treatment classes. World J. Diabetes.

[13] Palchuk M.B., London J.W., Perez-Rey D., Drebert Z.J., Winer-Jones J.P., Thompson C.N., Esposito J., Claerhout B. (2023). A global federated real-world data and analytics platform for research. JAMIA Open.

[14] Mankowski M.A., Bae S., Strauss A.T., Lonze B.E., Orandi B.J., Stewart D., Massie A.B., McAdams-DeMarco M.A., Oermann E.K., Habal M. (2025). Generalizability of kidney transplant data in electronic health records-The Epic Cosmos database vs the Scientific Registry of Transplant Recipients. Am. J. Transplant..

[15] Yen F.-S., Hsu C.-C., Yeh Y.-K., Cheng W.-Y., Liao P.-L., Hwu C.-M., Wei J.C.-C. (2025). The impact of sodium-glucose cotransporter-2 inhibitors on dialysis risk and mortality in kidney transplant patients with diabetes. Am. J. Transplant..

[16] Yen F., Hung Y., Huang J., Hsu C., Cheng W., Hwu C., Wei J.C. (2025). Effects of SGLT2 inhibitors on transplant survival and key clinical outcomes in heart transplant recipients with diabetes. J. Intern. Med..

[17] Lai G.-S., Li J.-R., Chen C.-S., Wang S.-S., Lin C.-Y., Yang C.-J., Ho H.-C., Hung S.-C., Chiu K.-Y., Yang C.-K. (2025). Temporal trends in malignancy incidence and outcomes among kidney transplantation recipients: A multi-center real-world evidence study using the TriNetX network (2000–2010 vs. 2010–2021). Front Immunol..

[18] Johnson J.C., Malik M., Engebretsen T.L., Mujtaba M., Lea A.S., Stevenson H.L., Kueht M.L. (2024). Assessing Long-Term Adverse Outcomes in Older Kidney Transplant Recipients: A Propensity Score-Matched Comparison of Early Steroid Withdrawal Versus Continuous Steroid Immunosuppression Using a Large Real-World Database. Drugs Aging.

[19] Publication Guidelines. https://trinetx.com/real-world-resources/case-studies-publications/trinetx-publication-guidelines/.

[20] von Elm E., Altman D.G., Egger M., Pocock S.J., Gotzsche P.C., Vandenbroucke J.P. (2007). The Strengthening the Reporting of Observational Studies in Epidemiology (STROBE) Statement: Guidelines for reporting observational studies. Lancet.

[21] Rana A., Murthy B., Pallister Z., Kueht M., Cotton R., Galvan N.T.N., Etheridge W., Liu H., Goss J., O’mahony C. (2017). Profiling risk for acute rejection in kidney transplantation: Recipient age is a robust risk factor. J. Nephrol..

[22] Cippà P.E., Schiesser M., Ekberg H., van Gelder T., Mueller N.J., Cao C.A., Fehr T., Bernasconi C. (2015). Risk stratification for rejection and infection after kidney transplantation. CJASN.

[23] Lebranchu Y., Baan C., Biancone L., Legendre C., Morales J.M., Naesens M., Thomusch O., Friend P. (2014). Pretransplant identification of acute rejection risk following kidney transplantation. Transpl. Int..

[24] Abbes S., Metjian A., Gray A., Martinu T., Snyder L., Chen D., Ellis M., Arepally G.M., Onwuemene O. (2017). HLA sensitization in solid organ transplantation: A primer on terminology, testing, and clinical significance for the apheresis practitioner. Ther. Apher. Dial..

[25] OPTN/UNOS Affected Policy Language. https://optn.transplant.hrsa.gov/media/2795/20190131_nlrb_policy.pdf.

[26] Ponticelli C., Reggiani F., Moroni G. (2022). Delayed Graft Function in Kidney Transplant: Risk Factors, Consequences and Prevention Strategies. J. Pers. Med..

[27] Cederborg A., Norén Å., Barten T., Lindkvist B., Bennet W., Herlenius G., Castedal M., Marschall H.-U., Åberg F. (2022). Renal function after liver transplantation: Real-world experience with basiliximab induction and delayed reduced-dose tacrolimus. Dig. Liver Dis..

[28] Ogata H., Aoki N., Nagano K., Hakamata M., Bamba Y., Shibata S., Koizumi T., Ohshima Y., Watanabe S., Moro H. (2022). Factors associated with cytomegalovirus antigenemia in patients with rheumatic disease: A retrospective study. J. Infect. Chemother..

