# Supplementary material for: Evaluating the Role of Basiliximab Induction in Simultaneous Liver–Kidney Transplantation: A Multicenter Propensity-Score-Matched Analysis
Source: Antibodies (Basel). 2025 Oct 28;14(4):91. doi: 10.3390/antib14040091 (PMC12641826; doi:10.3390/antib14040091)
Supplement: Supplementary file 1 [file antibodies-14-00091-s001.zip › Supplementary File S1, Codes.pdf]

Methods: Definition of Cohorts, 1:1 Propensity Score-Matching Criteria, and Outcomes Definitions

This study utilizes the TriNetX database, and this supplemental file includes the detailed definitions used in creating cohorts, for propensity score matching, and for outcome definitions.

1) Definition of Cohorts

Adult Liver Kidney Transplant Recipients

Adult (≥ 18 years old) recipients must have had a liver transplant procedure between December 31st, 2000 and December 31st, 2020 that was recorded in the EMR using the following codes:

1) CPT: 47135 Liver allotransplantation, orthotopic, partial or whole, from cadaver or living donor, any age (at least 18 years old at event)
2) ICD-10-PCS: 0FY00Z0 Transplantation of Liver, Allogeneic, Open Approach (at least 18 years old at event)

Recipients also must have had a kidney transplant procedure recorded in the EMR between 1 day before or 1 day after the liver transplant procedure.

1) ICD-10-PCS: 0TY10Z0 Transplantation of Left Kidney, Allogeneic, Open Approach (at least 18 years old at event)
2) CPT: 50360 Renal allotransplantation, implantation of graft; without recipient nephrectomy (at least 18 years old at event)
3) CPT: 1008109 Renal allotransplantation, implantation of graft (at least 18 years old at event)
4) CPT: 50365 Renal allotransplantation, implantation of graft; with recipient nephrectomy (at least 18 years old at event)
5) ICD-10-PCS :0TY00Z0 Transplantation of Right Kidney, Allogeneic, Open Approach (at least 18 years old at event)

Exclusion of other transplant procedures any time before the liver transplant procedure:

1) ICD-10-PCS: 02YA0Z0 Transplantation of Heart, Allogeneic, Open Approach
2) CPT: 1006332 Heart/Lung Transplantation Procedures
3) CPT: 1007944 Pancreas Transplantation Procedures
4) ICD10-CM: Z94.82 Intestine transplant status
5) ICD10-CM: Z94.81 Bone marrow transplant status

SLK recipients must have had an immunosuppression regimen of tac AND MMF or MMP AND prednisone recorded in the EMR within 6 months on or after the liver transplant procedure.

1) RXNORM:42316 tacrolimus
2) RXNORM:68149 mycophenolate mofetil
3) RXNORM:7145 mycophenolic acid
4) RXNORM:8640 prednisone

<u>Adult, Basiliximab (Bas) Cohort</u>

After the inclusion/exclusion on the previous codes, SLK recipients were selected into the Bas cohort if there was record of induction immunosuppression with Basiliximab in the EMR within 7 days on or after the renal transplant procedure.

1) RXNORM:196102 basiliximab

Recipients in the bas cohort was excluded if they had other induction immunosuppression agents recorded in the EMR within 7 days on or after the renal transplant procedure. Recipients that received steroids for induction were not excluded.

1) RXNORM:107044 Rabbit anti-human T-lymphocyte globulin (deprecated 2020)
2) RXNORM:117055 alemtuzumab
3) RXNORM:1011 lymphocyte immune globulin, anti-thymocyte globulin
4) RXNORM:91601 Lymphocyte immune globulin (deprecated 2020)
5) RXNORM:325523 LYMPHOCYTE IMMUNE GLOBULIN, RABBIT (deprecated 2020)

<u>Adult, No Basiliximab (No bas) Cohort</u>

Recipients in the No bas cohort were excluded if they received induction immunosuppression with basiliximab, anti-thymocyte globulin, or alemtuzumab within 7 days on or after transplant. Recipients were allowed to have received induction immunosuppression with steroids.

1) RXNORM:196102 basiliximab
2) RXNORM:107044 Rabbit anti-human T-lymphocyte globulin (deprecated 2020)
3) RXNORM:117055 alemtuzumab
4) RXNORM:1011 lymphocyte immune globulin, anti-thymocyte globulin
5) RXNORM:91601 Lymphocyte immune globulin (deprecated 2020)
6) RXNORM:325523 LYMPHOCYTE IMMUNE GLOBULIN, RABBIT (deprecated 2020)

1:1 Propensity Score Matching Criteria

Cohorts were matched on the following characteristics using 1:1 logistic regression model to balance cohorts proportionally:

1. Age- Age at Index
2. M- Male
3. 2054-5 Black or African American
4. 2135-2 Hispanic or Latino
5. 2186-5 Not Hispanic or Latino
6. 2106-3 White
7. 1002-5 American Indian or Alaska Native
8. 2028-9 Asian
9. 9083 BMI
10. E66- Overweight and obesity
11. B20 Human immunodeficiency virus (HIV)

12. B15-B19 Viral hepatitis
13. B19.2- Unspecified viral hepatitis C
14. B19.1- Unspecified viral hepatitis B
15. K70- Alcoholic liver disease
16. K76.0- Fatty (change of) liver, not elsewhere classified
17. K75.81- Nonalcoholic steatohepatitis
18. C22.0- Liver cell carcinoma
19. K74.3- Primary biliary cirrhosis
20. K83.01- Primary sclerosing cholangitis
21. K75.4- Autoimmune hepatitis
22. I10- Essential (primary) hypertension
23. E08-E13 Diabetes mellitus
24. M32- Systemic lupus erythematosus (SLE)
25. N03.3 Chronic nephritic syndrome with diffuse mesangial proliferative glomerulonephritis
26. N05.3 Unspecified nephritic syndrome with diffuse mesangial proliferative glomerulonephritis
27. N05.1 Unspecified nephritic syndrome with focal and segmental glomerular lesions
28. N04.1 Nephrotic syndrome with focal and segmental glomerular lesions
29. K76.7- Hepatorenal syndrome
30. K76.6 Portal hypertension
31. 0W9G30Z- Drainage of peritoneal cavity with drainage device, percutaneous approach
32. CPT 1020907 Abdominal paracentesis (diagnostic or therapeutic)
33. 0W9G3ZZ- Drainage of Peritoneal Cavity, Percutaneous Approach
34. K76.82- Hepatic encephalopathy
35. J96- Respiratory failure, not elsewhere classified
36. R57- Shock, not elsewhere classified
37. 1012752- Hemodialysis Procedures
38. 90945- Dialysis procedure other than hemodialysis (eg, peritoneal dialysis, hemofiltration, or other continuous renal replacement therapies), with single evaluation by a physician or other qualified health care professional
39. 1013729- Critical Care services
40. 44760-7 Model for end-stage liver disease score
41. 6963- Midodrine
42. 11149- Vasopressin (usp)
43. 7512- Norepinephrine
44. 8163- Phenylephrine
45. 828529- Albumin, usp
46. VT700- Vitamin K
47. 7617- Octreotide
48. 30243N1- Transfusion of Nonautologous Red Blood Cells into Central Vein, Percutaneous Approach
49. 30233N1- Transfusion of Nonautologous Red Blood Cells into Peripheral Vein, Percutaneous Approach
50. 36430- Transfusion, blood or blood components

51. 9029- Sodium [moles/volume] in Serum, Plasma, or Blood
52. 9050- Bilirubin, total [mass/volume] in Serum, Plasma, or Blood
53. 9020- Platelets [#/volume] in Blood
54. 9032- INR in Plasma or Blood
55. 9045- Albumin [mass/volume]
56. 9024- Creatinine [mass/volume]
57. 5124-3 Cytomegalovirus IgG Ab [Units/volume] in Serum or Plasma by Immunoassay
58. 30339-6 Epstein Barr virus capsid IgG Ab [Presence] in Serum
59. 19162-7 Varicella zoster virus IgG Ab [Presence] in Serum
60. 6A550Z3- Pheresis of plasma, single
61. 36514- Therapeutic apheresis; for plasma pheresis
62. Transfusion of Red Blood Cells
63. Transfusion, blood or components
64. 34960-5- HLA Ab [Identifier] in Serum or Plasma
65. 50968-7- HLA Ab [Type] in Serum
66. 40734-6- HLA Ab [Units/volume] in Serum
67. 44534-6- HLA Ab [Presence] in Serum
68. 41618-0- HLA Ab [Presence] in Serum by Flow cytometry (FC)
69. 77640-1- HLA class I and II IgG Ab in Serum or Plasma by Immunoassay [interpretation] Narrative
70. 807370- Calculated panel reactive antibody- Serum
71. 10- Pregnancy
72. T86.41 Liver transplant rejection
73. T86.12 Kidney transplant failure
74. T86.11 Kidney transplant rejection
75. T86.42 Liver transplant failure
76. 1007820 Backbench standard preparation of cadaver donor whole liver graft prior to allotransplantation, including cholecystectomy, if necessary, and dissection and removal of surrounding soft tissues to prepare the vena cava, portal vein, hepatic artery, and common bile duct for implantation
77. 1008104 Backbench reconstruction of cadaver or living donor renal allograft prior to transplantation
78. 50323 Backbench standard preparation of cadaver donor renal allograft prior to transplantation, including dissection and removal of perinephric fat, diaphragmatic and retroperitoneal attachments, excision of adrenal gland, and preparation of ureter(s), renal vein(s), and renal artery(s), ligating branches, as necessary
79. 50325 Backbench standard preparation of living donor renal allograft (open or laparoscopic) prior to transplantation, including dissection and removal of perinephric fat and preparation of ureter(s), renal vein(s), and renal artery(s), ligating branches, as necessary
80. 0FY00Z0 Transplantation of Liver, Allogeneic, Open Approach
81. 1007811 Liver Transplantation Procedures
82. 0TY00Z0 Transplantation of Right Kidney, Allogeneic, Open Approach
83. 0TY10Z0 Transplantation of Left Kidney, Allogeneic, Open Approach

Outcomes Definitions

1) Liver or kidney transplant rejection: defined as treated or diagnosed for rejection
    a. UMLS:ICD10CM:T86.41, Liver transplant rejection
    b. UMLS:ICD10CM:T86.11, Kidney transplant rejection
    c. NLM:RXNORM:1011, lymphocyte immune globulin, anti-thymocyte globulin
    d. NLM:RXNORM:6902, methylprednisolone (Strength: 100 MG or 125 MG or 500 MG or 1000 MG or 2000 MG)
    e. UMLS:HCPCS:J1459, Injection, immune globulin (privigen), intravenous, non-lyophilized (e.g., liquid), 500 mg
    f. UMLS:HCPCS:J1556, Injection, immune globulin (bivigam), 500 mg
    g. UMLS:HCPCS:J1557, Injection, immune globulin, (gammaplex), intravenous, non-lyophilized (e.g., liquid), 500 mg
    h. UMLS:HCPCS:J1561, Injection, immune globulin, (gamunex-c/gammaked), non-lyophilized (e.g., liquid), 500 mg
    i. UMLS:HCPCS:J1566, Injection, immune globulin, intravenous, lyophilized (e.g., powder), not otherwise specified, 500 mg
    j. UMLS:HCPCS:J1568, Injection, immune globulin, (octagam), intravenous, non-lyophilized (e.g., liquid), 500 mg
    k. UMLS:HCPCS:J1569, Injection, immune globulin, (gammagard liquid), non-lyophilized, (e.g., liquid), 500 mg
    l. UMLS:HCPCS:J1572, Injection, immune globulin, (flebogamma/flebogamma dif), intravenous, non-lyophilized (e.g., liquid), 500 mg
    m. UMLS:HCPCS:J1599, Injection, immune globulin, intravenous, non-lyophilized (e.g., liquid), not otherwise specified, 500 mg

2) Liver transplant rejection, defined as diagnosed as rejection
    a. UMLS:ICD10CM:T86.41, Liver transplant rejection

3) Kidney transplant rejection, defined as diagnosed as rejection
    a. UMLS:ICD10CM:T86.11, Kidney transplant rejection

4) Liver biopsy
    a. UMLS:ICD10PCS:0FB03ZX, Excision of Liver, Percutaneous Approach, Diagnostic
    b. UMLS:ICD10PCS:0FB04ZX, Excision of Liver, Percutaneous Endoscopic Approach, Diagnostic
    c. UMLS:CPT:1007797, Biopsy of liver, needle
    d. UMLS:CPT:47001, Biopsy of liver, needle; when done for indicated purpose at time of other major procedure (List separately in addition to code for primary procedure)
    e. UMLS:CPT:47100, Biopsy of liver, wedge
    f. UMLS:SNOMED:274325001, Percutaneous liver biopsy
    g. UMLS:SNOMED:86259008, Biopsy of liver

5) Kidney biopsy
    a. UMLS:SNOMED:7246002, Kidney biopsy
    b. UMLS:CPT:50205, Renal biopsy; by surgical exposure of kidney
    c. UMLS:CPT:50200, Renal biopsy; percutaneous, by trocar or needle
    d. UMLS:ICD10PCS:0TB13ZX, Excision of Left Kidney, Percutaneous Approach, Diagnostic
    e. UMLS:ICD10PCS:0TB03ZX, Excision of Right Kidney, Percutaneous Approach, Diagnostic
    f. UMLS:ICD10PCS:0TB14ZX, Excision of Left Kidney, Percutaneous Endoscopic Approach, Diagnostic
    g. UMLS:ICD10PCS:0TB43ZX, Excision of Left Kidney Pelvis, Percutaneous Approach, Diagnostic
    h. UMLS:ICD10PCS:0TB04ZX, Excision of Right Kidney, Percutaneous Endoscopic Approach, Diagnostic

6) Dialysis
    a. CPT:1012740, Dialysis Services and Procedures
    b. CPT:90945, Dialysis procedure other than hemodialysis (eg, peritoneal dialysis, hemofiltration, or other continuous renal replacement therapies), with single evaluation by a physician or other qualified health care professional
    c. CPT:90947, Dialysis procedure other than hemodialysis (eg, peritoneal dialysis, hemofiltration, or other continuous renal replacement therapies) requiring repeated evaluations by a physician or other qualified health care professional, with or without substantial revision of dialysis prescription
    d. UMLS:CPT:90935 Hemodialysis procedure with single evaluation by a physician or other qualified health care professional
    e. UMLS:ICD9CM:39.95 Hemodialysis
    f. UMLS:CPT:90937 Hemodialysis procedure requiring repeated evaluation(s) with or without substantial revision of dialysis prescription
    g. UMLS:CPT:1012752 Hemodialysis Procedures

7) Hemodialysis
    a. UMLS:CPT:90935 Hemodialysis procedure with single evaluation by a physician or other qualified health care professional
    b. UMLS:ICD9CM:39.95 Hemodialysis
    c. UMLS:CPT:90937 Hemodialysis procedure requiring repeated evaluation(s) with or without substantial revision of dialysis prescription
    d. UMLS:CPT:1012752 Hemodialysis Procedures

8) Delayed graft function – dialysis or hemodialysis within 7 days post-transplant (2-8 days after index event; index event was defined as liver transplant procedure, kidney transplant procedure was defined as within 1 day before or after liver transplantation procedure.)
    a. Previously described codes for dialysis
    b. Previously described codes for hemodialysis

9) Liver re-transplant
    a. ICD10PCS:0FY00Z0, Transplantation of Liver, Allogeneic, Open Approach
    b. CPT:47143, Backbench standard preparation of cadaver donor whole liver graft prior to allotransplantation, including cholecystectomy, if necessary, and dissection and removal of surrounding soft tissues to prepare the vena cava, portal vein, hepatic artery, and common bile duct for implantation; without trisegment or lobe split

10) Kidney graft failure, defined as eGFR ≤15 mL/min/{1.73_m2}
    a. UMLS:LNC:62238-1, Glomerular filtration rate/1.73 sq M.predicted [Volume Rate/Area] in Serum, Plasma or Blood by Creatinine-based formula (CKD-EPI) (at most 15.00 mL/min/{1.73_m2} (most recent occurrence))

11) Mortality: defined as TriNetX demographic data as deceased
    a. Deceased

12) Hospitalizations
    a. UMLS:CPT:1013659, Hospital Inpatient and Observation Care Services
    b. UMLS:CPT:1013675, Hospital Inpatient or Observation Care Services (Including Admission and Discharge Services)

13) CMV Viremia: defined as diagnosed cytomegaloviral disease or laboratory confirmed CMV viral load (at least 1000.00 {copies}/mL or at least 3.00 {Log_IU}/mL)
    a. UMLS: LNC:30247-1, Cytomegalovirus DNA [#/volume] (viral load) in Serum or Plasma by NAA with probe detection (at least 1000.00 {copies}/mL (most recent occurrence))
    b. UMLS: LNC:29604-6, Cytomegalovirus DNA [#/volume] (viral load) in Blood by NAA with probe detection (at least 1000.00 {copies}/mL (most recent occurrence))
    c. UMLS: LNC:72493-0, Cytomegalovirus DNA [Units/volume] (viral load) in Plasma by NAA with probe detection (at least 1000.00 [IU]/mL (most recent occurrence))
    d. UMLS: LNC:33006-8, Cytomegalovirus DNA [#/volume] (viral load) in Specimen by NAA with probe detection (at least 1000.00 {copies}/mL (most recent occurrence))
    e. UMLS: LNC:34720-3, Cytomegalovirus DNA [Units/volume] (viral load) in Specimen by NAA with probe detection (at least 1000.00 [IU]/mL (most recent occurrence))
    f. UMLS: LNC:24041-6, Cytomegalovirus DNA [Units/volume] (viral load) in Specimen by Probe with signal amplification (at least 1000.00 [arb'U]/mL (most recent occurrence))
    g. UMLS: LNC:49347-8, Cytomegalovirus DNA [#/volume] (viral load) in Urine by NAA with probe detection (at least 1000.00 {copies}/mL (most recent occurrence))
    h. UMLS: LNC:49351-0, Cytomegalovirus DNA [#/volume] (viral load) in Tissue by NAA with probe detection (at least 1000.00 {copies}/mL (most recent occurrence))
    i. UMLS: LNC:54206-8, Cytomegalovirus DNA [Log #/volume] (viral load) in Serum or Plasma by NAA with probe detection (at least 3.00 {Log_copies}/mL (most recent occurrence))

j. UMLS: LNC:53763-9, Cytomegalovirus DNA [Log #/volume] (viral load) in Specimen by NAA with probe detection (at least 3.00 {Log_copies}/mL (most recent occurrence))
k. UMLS: LNC:72494-8, Cytomegalovirus DNA [log units/volume] (viral load) in Plasma by NAA with probe detection (at least 3.00 {Log_IU}/mL (most recent occurrence))
l. UMLS: LNC:96396-7, Cytomegalovirus DNA [log units/volume] (viral load) in Specimen by NAA with probe detection (at least 3.00 {Log_IU}/mL (most recent occurrence))
m. UMLS:ICD10CM:B25, Cytomegaloviral disease

14) EBV viremia: defined as diagnosed infectious mononucleosis or laboratory confirmed EBV viral load (at least 1000.00 {copies}/mL or at least 3.00 {Log_IU}/mL)
    a. UMLS: LNC:32585-2, Epstein Barr virus DNA [#/volume] (viral load) in Specimen by NAA with probe detection (at least 1000.00 {copies}/mL (most recent occurrence))
    b. UMLS: LNC:53774-6, Epstein Barr virus DNA [Log #/volume] (viral load) in Specimen by NAA with probe detection (at least 3.00 {Log_copies}/mL (most recent occurrence))
    c. UMLS: LNC:36923-1, Epstein Barr virus DNA [#/volume] (viral load) in Blood by NAA with probe detection (at least 1000.00 {copies}/mL (most recent occurrence))
    d. UMLS: LNC:93840-7, Epstein Barr virus DNA [Units/volume] (viral load) in Blood by NAA with probe detection (at least 1000.00 [IU]/mL (most recent occurrence))
    e. UMLS: LNC:93841-5, Epstein Barr virus DNA [log units/volume] (viral load) in Blood by NAA with probe detection (at least 3.00 {Log_IU}/mL (most recent occurrence))
    f. UMLS: LNC:43730-1, Epstein Barr virus DNA [Units/volume] (viral load) in Serum or Plasma by NAA with probe detection (at least 1000.00 [IU]/mL (most recent occurrence))
    g. UMLS: LNC:47982-4, Epstein Barr virus DNA [#/volume] (viral load) in Serum or Plasma by NAA with probe detection (at least 1000.00 {copies}/mL (most recent occurrence))
    h. UMLS:ICD10CM:B27.9, Infectious mononucleosis, unspecified

15) BK viremia: defined as laboratory confirmed BK viral load (at least 1000.00 {copies}/mL or at least 3.00 {Log_IU}/mL)
    a. UMLS:LNC:41479-7, BK virus DNA [#/volume] (viral load) in Serum or Plasma by NAA with probe detection (at least 1000.00 {copies}/uL (most recent occurrence))
    b. UMLS:LNC:41480-5, BK virus DNA [#/volume] (viral load) in Urine by NAA with probe detection (at least 1000.00 {copies}/uL (most recent occurrence))
    c. UMLS:LNC:48309-9, BK virus DNA [#/volume] (viral load) in Specimen by NAA with probe detection (at least 1000.00 {copies}/mL (most recent occurrence))
    d. UMLS:LNC:32284-2, BK virus DNA [Units/volume] (viral load) in Serum or Plasma by NAA with probe detection (at least 1000.00 [IU]/mL (most recent occurrence))

e. UMLS:LNC:49345-2, BK virus DNA [#/volume] (viral load) in Blood by NAA with probe detection (at least 1000.00 {copies}/mL (most recent occurrence))
f. UMLS:LNC:32285-9, BK virus DNA [Units/volume] (viral load) in Urine by NAA with probe detection (at least 1000.00 [IU]/mL (most recent occurrence))
g. UMLS:LNC:49344-5, BK virus DNA [#/volume] (viral load) in Cerebral spinal fluid by NAA with probe detection (at least 1000.00 {copies}/mL (most recent occurrence))
h. UMLS:LNC:42587-6, BK virus DNA [Units/volume] (viral load) in Specimen by NAA with probe detection (at least 1000.00 [arb'U]/mL (most recent occurrence))
i. UMLS:LNC:43201-3, BK virus DNA [Log #/volume] (viral load) in Specimen by NAA with probe detection (at least 3.00 {Log_copies}/mL (most recent occurrence))
j. UMLS:LNC:90924-2, BK virus DNA [Log #/volume] (viral load) in Urine by NAA with probe detection (at least 3.00 {Log_copies}/mL (most recent occurrence))
k. UMLS:LNC:44805-0, BK virus DNA [Log #/volume] (viral load) in Serum or Plasma by NAA with probe detection (at least 3.00 {Log_copies}/mL (most recent occurrence))

16) JC viremia: defined as laboratory confirmed JC viral load (at least 1000.00 {copies}/mL or at least 3.00 {Log_IU}/mL) or diagnosed progressive multifocal leukoencephalopathy
    a. UMLS:LNC:49412-0, JC virus DNA [#/volume] (viral load) in Specimen by NAA with probe detection (at least 1000.00 {copies}/mL (most recent occurrence))
    b. UMLS:LNC:49414-6, JC virus DNA [#/volume] (viral load) in Blood by NAA with probe detection (at least 1000.00 {copies}/mL (most recent occurrence))
    c. UMLS:LNC:49411-2, JC virus DNA [#/volume] (viral load) in Urine by NAA with probe detection (at least 1000.00 {copies}/mL (most recent occurrence))
    d. UMLS:LNC:49545-7, JC virus DNA [Units/volume] (viral load) in Cerebral spinal fluid by NAA with probe detection (at least 1000.00 [arb'U]/mL (most recent occurrence))
    e. UMLS:LNC:49410-4, JC virus DNA [#/volume] (viral load) in Cerebral spinal fluid by NAA with probe detection (at least 1000.00 {copies}/mL (most recent occurrence))
    f. UMLS:LNC:49413-8, JC virus DNA [#/volume] (viral load) in Serum or Plasma by NAA with probe detection (at least 1000.00 {copies}/mL (most recent occurrence))
    g. UMLS:LNC:100685-7, JC virus DNA [Log #/volume] (viral load) in Serum or Plasma by NAA with probe detection (at least 3.00 {Log_copies}/mL (most recent occurrence))
    h. ICD10CM:A81.2 – Progressive multifocal leukoencephalopathy

17) VZV Viremia: defined as diagnosed varicella (herpes zoster) infection or laboratory confirmed VZV viral load (at least 1000.00 {copies}/mL)
    a. ICD10CM:B02 - Zoster [herpes zoster]
    b. LNC:49451-8 - Varicella zoster virus DNA [#/volume] (viral load) in Specimen by NAA with probe detection (at least 1000.00 {copies}/mL (most recent occurrence))
    c. LNC:49455-9 - Varicella zoster virus DNA [#/volume] (viral load) in Bronchoalveolar lavage by NAA with probe detection (at least 1000.00 {copies}/mL (most recent occurrence))

d. LNC:47003-9 - Varicella zoster virus DNA [#/volume] (viral load) in Serum or Plasma by NAA with probe detection (at least 1000.00 {copies}/mL (most recent occurrence))
e. LNC:47002-1 - Varicella zoster virus DNA [#/volume] (viral load) in Cerebral spinal fluid by NAA with probe detection (at least 1000.00 {copies}/mL (most recent occurrence))

18) Composite viremia: composite of diagnosed or laboratory confirmed viral load (load (at least 1000.00 {copies}/mL or at least 3.00 {Log_IU}/mL)
  a. All previously described CMV viremia codes
  b. All previously described EBV viremia codes
  c. All previously described BK viremia codes
  d. All previously described JC viremia codes
  e. All previously described VZV viremia codes

19) Sepsis: defined as diagnosis of sepsis
  a. UMLS:ICD10CM:A41, Other sepsis
  b. UMLS:ICD10CM:A40, Streptococcal sepsis
  c. UMLS:ICD10CM:R65.2, Severe sepsis

20) Viral, bacterial , or other infectious pneumonia: defined as diagnosis of pneumonia
  a. UMLS:ICD10CM:J18, Pneumonia, unspecified organism
  b. UMLS:ICD10CM:J15, Bacterial pneumonia, not elsewhere classified
  c. UMLS:ICD10CM:J16, Pneumonia due to other infectious organisms, not elsewhere classified
  d. UMLS:ICD10CM:J17, Pneumonia in diseases classified elsewhere
  e. UMLS:ICD10CM:J14, Pneumonia due to Hemophilus influenzae
  f. UMLS:ICD10CM:J13, Pneumonia due to Streptococcus pneumoniae
  g. UMLS:ICD10CM:J12, Viral pneumonia, not elsewhere classified

21) Pyelonephritis: defined as diagnosed pyelonephritis or kidney transplant infection
  a. UMLS:ICD10CM:N10, Acute pyelonephritis
  b. UMLS:ICD10CM:T86.13, Kidney transplant infection

Descriptive Outcomes

1) eGFR: CKD-EPI measured at 14 days, 3 months, 6 months, and 1 year post-transplant
  a. UMLS:LNC:62238-1, Glomerular filtration rate/1.73 sq M.predicted [Volume Rate/Area] in Serum, Plasma or Blood by Creatinine-based formula (CKD-EPI) (most recent occurrence)

2) AST: measured at 14 days, 3 months, 6 months, and 1 year post-transplant
  a. TNX:9047 Aspartate aminotransferase [Enzymatic activity/volume] in Serum or Plasma ((most recent occurrence))

3) ALT: measured at 14 days, 3 months, 6 months, and 1 year post-transplant
    a. TNX:9044 Alanine aminotransferase [Enzymatic activity/volume] in Serum, Plasma or Blood ((most recent occurrence))

4) INR: measured at 14 days, 3 months, 6 months, and 1 year post-transplant
    a. TNX:9032 INR in Plasma or Blood ((most recent occurrence))

5) Total bilirubin: measured at 14 days, 3 months, 6 months, and 1 year post-transplant
    a. TNX:9050 Bilirubin.total [Mass/volume] in Serum, Plasma or Blood ((most recent occurrence))

6) Tacrolimus trough level: measured at 14 days, 3 months, 6 months, and 1-year post-transplant
    a. 11253-2: Tacrolimus [Mass/volume] in Blood ((most recent occurrence))
