# Supplementary material for: Evaluating the Role of Basiliximab Induction in Simultaneous Liver–Kidney Transplantation: A Multicenter Propensity-Score-Matched Analysis
Source: Antibodies (Basel). 2025 Oct 28;14(4):91. doi: 10.3390/antib14040091 (PMC12641826; doi:10.3390/antib14040091)
Supplement: Supplementary file 1 [file antibodies-14-00091-s001.zip › Supplementary Table S2, Propensity-Score Matching Results.pdf]

Supplemental Results

Table 1. Propensity Score Matching Results. Results are listed as mean ± standard deviation (SD) for characteristics with numeric values, such as age, BMI, and lab values, and as the absolute number of individuals (n) with percent of cohort (%) recorded for other descriptive characteristics such as gender, race/ethnicity, and diagnoses.

	**Cohorts, prior to propensity-score matching (mean +/- SD; % cohort (n))**			**Cohorts, after propensity-score matching (mean +/- SD; % cohort (n))**		
	**Bas**	**No Bas**	**p value**	**Bas**	**No Bas**	**p value**
**Demographics**						
Cohort size	365	772	N/A	292	292	N/A
Age	56.7 ± 10.0	56.7 ± 10.7	0.97	56.7 ± 9.9	57.2 ± 10.2	0.62
Male	231 (63.0%)	417 (56.8%)	0.05	174 (59.7%)	183 (62.8%)	0.44
Black or African American	69 (18.9%)	87 (12.2%)	0.003	52 (17.7%)	43 (14.6%)	0.31
Hispanic or Latino	18 (4.9%)	107 (14.7%)	<0.001	17 (5.9%)	29 (10.1%)	0.07
White	244 (66.3%)	534 (72.2%)	0.04	193 (66.3%)	200 (68.4%)	0.60
American Indian or Alaska Native	10 (2.7%)	10 (1.7%)	0.24	10 (3.5%)	10 (3.5%)	1
Asian	12 (3.3%)	15 (1.9%)	0.17	10 (3.5%)	10 (3.5%)	1
**Diagnoses**						
BMI	28.0±6.3	28.3±6.0	0.47	28.4±6.3	28.0±5.9	0.54
Overweight and obesity	130 (36.4%)	269 (37.9%)	0.63	105 (35.8%)	109 (37.2%)	0.73
Human immunodeficiency virus (HIV)	10 (2.7%)	10 (1.3%)	0.09	10 (3.5%)	10 (3.5%)	1
**Etiologies of liver disease**						
Viral hepatitis	112 (32.1%)	251 (35.1%)	0.32	91 (31.3%)	88 (30.2%)	0.79
Unspecified viral hepatitis C	86 (24.1%)	180 (25.2%)	0.69	70 (24.0%)	68 (23.3%)	0.84
Unspecified viral hepatitis B	19 (5.5%)	22 (3.5%)	0.12	14 (4.9%)	10 (3.5%)	0.40
Alcoholic liver disease	191 (52.3%)	374 (50.6 %)	0.60	148 (50.7%)	146 (50.0%)	0.87

Fatty liver	76 (21.9%)	183 (25.3%)	0.21	67 (22.9%)	75 (25.7%)	0.44
Nonalcoholic steatohepatitis	116 (32.9%)	235 (32.2%)	0.82	103 (35.4%)	89 (30.6%)	0.22
Liver cell carcinoma	46 (12.9%)	106 (15.2%)	0.30	39 (13.2%)	37 (12.8%)	0.90
Primary biliary cirrhosis	20 (5.8%)	44 (6.5%)	0.63	15 (5.2%)	13 (4.5%)	0.70
Primary sclerosing cholangitis	10 (2.7%)	18 (2.2%)	0.58	10 (3.5%)	10 (3.5%)	1
Autoimmune hepatitis	10 (2.7%)	30 (4.8%)	0.10	10 (3.5%)	10 (3.5%)	1
**Etiologies of kidney disease**						
Primary hypertension	226 (63.8%)	457 (64.0%)	0.95	187 (63.9%)	187 (64.2%)	0.93
Diabetes mellitus	184 (51.8%)	398 (56.9%)	0.11	148 (50.7%)	149 (51.0%)	0.93
Systemic lupus erythematosus (SLE)	10 (2.7%)	10 (1.7%)	0.24	10 (3.5%)	10 (3.5%)	1
Chronic nephritic syndrome with diffuse mesangial proliferative glomerulonephritis	10 (2.7%)	10 (2.6%)	0.88	0 (0.0%)	0 (0.0%)	N/A
Nephritic syndrome with focal and segmental glomerular lesions	10 (2.7%)	10 (2.6%)	0.89	10 (3.5%)	10 (3.5%)	1
Hepatorenal syndrome	171 (50.1%)	385 (55.6%)	0.09	148 (50.7%)	146 (50.0%)	0.87
**Illness Severity**						
Portal hypertension	279 (78.9%)	568 (77.3%)	0.54	225 (77.1%)	217 (74.3%)	0.44
Abdominal paracentesis	176 (48.2%)	357 (49.5%)	0.69	137 (46.9%)	143 (49.0%)	0.62
Hepatic encephalopathy	89 (24.4%)	261 (34.0%)	0.001	78 (26.7%)	73 (25.0%)	0.63
Respiratory failure	121 (36.7%)	242 (36.4%)	0.91	97 (33.3%)	97 (33.7%)	0.93
Shock	82 (22.5%)	216 (28.1%)	0.05	64 (21.9%)	70 (24.0%)	0.55
Hemodialysis	136 (37.3%)	399 (51.8%)	<0.001	110 (37.8%)	119 (40.6%)	0.50

Peritoneal dialysis, CRRT, hemofiltration	87 (24.1%)	181 (23.5%)	0.82	63 (21.5%)	53 (18.1%)	0.30
Critical Care services	147 (40.5%)	341 (44.2%)	0.25	110 (37.8%)	114 (38.9%)	0.80
Model for end-stage liver disease score (n, % with value)	37.9 ± 8.2 (40, 10.9%)	36.4 ± 8.0 (27, 3.5%)	0.65	37.9 ± 8.2 (17, 5.9%)	36.4 ± 8.0 (10, 3.5%)	0.65
**Medications**						
Midodrine	192 (52.9%)	384 (53.1%)	0.94	146 (50.0%)	149 (51.0%)	0.80
Vasopressin	59 (16.2%)	222 (28.7%)	<0.001	48 (16.3%)	54 (18.4%)	0.51
Norepinephrine	73 (20.0%)	253 (32.7%)	<0.001	61 (20.8%)	65 (22.2%)	0.69
Phenylephrine	94 (26.0%)	221 (28.6%)	0.37	77 (26.4%)	73 (25.0%)	0.70
Albumin	255 (70.7%)	535 (69.6%)	0.71	198 (67.7%)	208 (71.2%)	0.37
Vitamin K	131 (36.2%)	232 (30.0%)	0.04	96 (33.0%)	97 (31.3%)	0.66
Octreotide	155 (42.5%)	310 (40.1%)	0.46	116 (39.9%)	118 (40.3%)	0.93
**Labs**						
Sodium [moles/volume]	135.7 ± 4.8	135.4 ± 4.6	0.39	135.5 ± 5.0	135.3 ± 4.8	0.59
Bilirubin, total [mass/volume]	5.9 ± 9.3	5.7 ± 9.1	0.72	5.0 ± 8.2	6.1 ± 10.1	0.20
Platelets [#/volume]	89.0 ± 67.9	86.5 ± 66.8	0.57	90.4 ± 66.4	92.3 ± 66.5	0.74
INR in Plasma or Blood	1.6 ± 0.6	1.6 ± 0.6	0.59	1.6 ± 0.6	1.6 ± 0.7	0.09
Albumin [mass/volume]	3.1 ± 0.7	3.0 ± 0.7	0.03	3.1 ± 0.7	3.1 ± 0.7	0.85
Creatinine [mass/volume]	3.8 ± 2.7	3.6 ± 3.9	0.41	4.0 ± 2.9	3.7 ± 2.6	0.25
Cytomegalovirus IgG Ab [units/volume] in Serum or Plasma (n, % cohort)	18.1 ± 63.2 (22, 6.3%)	29.9 ± 81.5 (100, 13.4%)	0.52	5.4 ± 12.3 (21, 7.3%)	26.5 ± 79.1 (27, 9.4%)	0.23
Varicella zoster virus IgG Ab [Presence] in Serum	10 (2.7%)	58 (7.5%)	0.47	10 (3.5%)	21 (7.3%)	0.50

Epstein Barr virus capsid IgG Ab [Presence] in Serum	10 (2.7%)	67 (8.7%)	0.58	10 (3.5%)	14 (4.9%)	0.40
**Immunologic risk**						
Plasmapheresis	10 (2.7%)	10 (1.3%)	0.09	10 (3.5%)	10 (3.5%)	1
Transfusion of Red Blood Cells	68 (18.9%)	232 (30.3%)	<0.001	58 (19.8%)	43 (14.6%)	0.20
Transfusion, blood or components	63 (17.5%)	201 (26.6%)	0.001	44 (14.9%)	44 (14.9%)	1
HLA Ab in Serum by Flow cytometry (FC) (n, % with value)	3.0 +/- 5.8 (40, 11.2%)	1.8 +/- 4.0 (10, 1.3%)	0.47	0.4 +/- 0.5 (17, 5.9%)	0.3 +/- 0.5 (10, 3.5%)	0.26
Pregnancy	10 (2.74%)	10 (1.30%)	0.08	10 (3.47%)	10 (3.47%)	1
**Graft Type**						
Backbench preparation of **living** donor renal allograft	10 (2.84%)	10 (1.30%)	0.07	11 (3.91%)	11 (3.91%)	1
Backbench preparation of **deceased** donor renal allograft	270 (74.43%)	605 (78.20%)	0.16	217 (74.22 %)	217 (80.01%)	0.11
Backbench preparation of **deceased** whole liver graft	284 (75.85%)	613 (79.24%)	0.20	217 (74.44 %)	243 (83.20%)	0.06
Backbench preparation of **deceased or living** renal allograft	47 (13.64%)	146 (18.93%)	0.03	38 (12.89 %)	56 (19.14%)	0.05
**Previous liver or kidney transplant**						
Liver transplant	10 (3.8%)	23 (3.8%)	1	11 (3.8%)	11 (3.8%)	1
Kidney transplant	0 (0%)	10 (1.3%)	0.03	0 (0%)	0 (0%)	1
Kidney transplant rejection diagnosis	22 (6.2%)	48 (6.3%)	0.80	14 (4.9%)	12 (4.1%)	0.68
Kidney transplant failure diagnosis	22 (6.2%)	48 (6.3%)	0.97	16 (5.6%)	15 (5.3%)	0.85
Liver transplant rejection diagnosis	14 (4.0%)	60 (7.8%)	0.02	13 (4.5%)	13 (4.5%)	1

Liver transplant failure diagnosis	15 (4.3%)	51 (6.6%)	0.16	14 (4.9%)	14 (4.5%)	0.84
